# Management of Innominate Artery Occlusion With Severe Left Common Carotid Artery Stenosis

**DOI:** 10.7759/cureus.19592

**Published:** 2021-11-15

**Authors:** Justin M George, Peter V Cooke, Nicole Ilonzo, Rami O Tadros, Robert J Grossi

**Affiliations:** 1 Division of Vascular Surgery, Department of Surgery, Icahn School of Medicine at Mount Sinai, New York, USA; 2 Division of Vascular and Endovascular Surgery, Department of Surgery, Weill Cornell, New York, USA

**Keywords:** vertebrobasilar insufficiency, subclavian steal syndrome, carotid-carotid bypass, supra-aortic trunk, innominate

## Abstract

Innominate artery occlusion is a rare entity, particularly when coupled with severe left common carotid artery stenosis. Innominate artery disease may present with varying degrees of symptomatology and can place patients at risk for both posterior fossa and hemispheric ischemic events. We present a symptomatic case of innominate artery occlusion with severe left common carotid disease. We reviewed the literature and current options for the treatment of innominate artery disease. The patient underwent successful hybrid repair with left carotid artery retrograde stenting and left carotid artery to right carotid artery bypass. She has been symptom and re-intervention free during her one-year follow-up. We describe a successful hybrid repair of symptomatic innominate artery occlusion with concomitant severe left carotid artery stenosis in a patient with a prohibitive open thoracic surgical risk.

## Introduction

Innominate artery stenosis or occlusion is a rare clinical presentation, far less common than subclavian artery disease. Radiographic findings of innominate artery stenosis comprise only 2.5% to 4% of atherosclerotic lesions of the extracranial cerebral arteries; however, this includes low-grade, likely asymptomatic stenosis [1,2]. The true incidence of symptomatic innominate artery disease is unknown. Up to 8% of patients who present with suspected subclavian steal syndrome in fact have an innominate artery lesion [3].

Innominate artery disease places patients at risk for both posterior fossa and hemispheric events including vertebrobasilar insufficiency, amaurosis fugax, and stroke [4]. Multiple clinical series have demonstrated that severe innominate artery lesions more dramatically alter right vertebral arterial flow versus right carotid arterial flow [2,5]. With an innominate artery occlusion, flow is siphoned from the basilar axis by the right vertebral artery and passed into the subclavian artery, where it continues retrograde into the right common carotid artery and then cephalad [3]. Canine models have demonstrated the preservation of cephalad flow in the carotid artery with the reversed right vertebral flow in innominate artery occlusion [6]. Midsystolic decelerations and elevated left common carotid artery to right common carotid artery peak systolic velocity ratios can be seen with innominate artery disease, though the degree of stenosis does not correlate with the degree of these duplex findings [4].

Below, the diagnosis and surgical management of a 72-year-old female presenting with vertebrobasilar insufficiency due to innominate artery occlusion with severe left common carotid artery insufficiency is described.

## Case presentation

**Figure 1:**
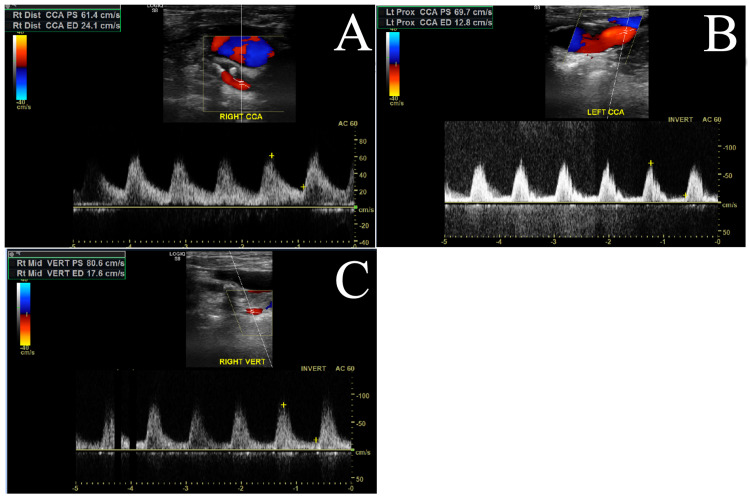
Preoperative duplex of the right common carotid artery (A), left common carotid artery (B), and right vertebral artery (C). Note the persistent retrograde flow in the right vertebral artery throughout the cardiac cycle.

A 72-year-old female with a history of hypertension, diabetes, coronary artery disease with a history of coronary stents, and squamous cell carcinoma of the tongue treated with a hemiglossectomy, neck exploration, and free flap reconstruction presented with increasingly frequent syncopal episodes most likely due to vertebrobasilar insufficiency. The diagnostic workup included carotid and vertebral artery duplex imaging, which demonstrated normal bilateral carotid waveforms (Figure 1A, 1B), normal left vertebral artery waveform, but a sustained reversal of flow in the right vertebral artery (Figure 1C).

**Figure 2:**
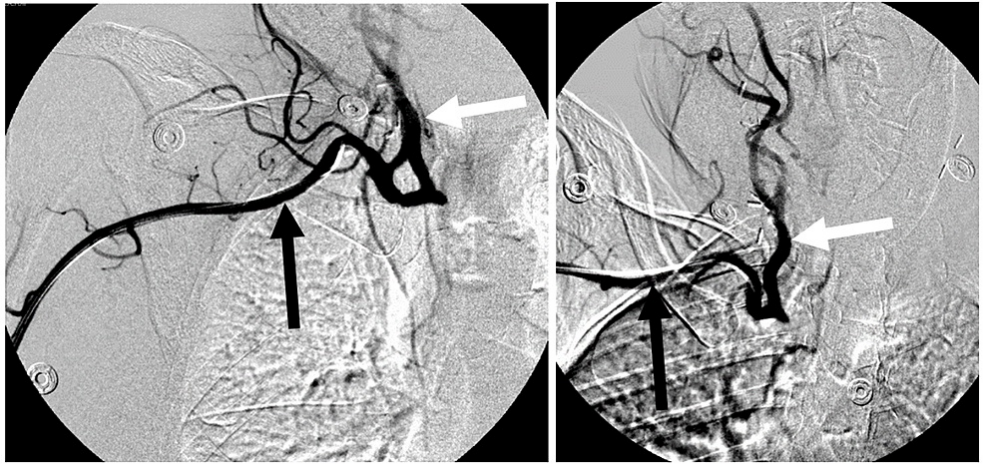
Preoperative right transradial angiogram demonstrating innominate occlusion with filling of the right subclavian (black arrow) and the right common carotid (white arrow) arteries.

Transradial subclavian angiogram demonstrated complete occlusion of the innominate artery (Figure 2).

**Figure 3:**
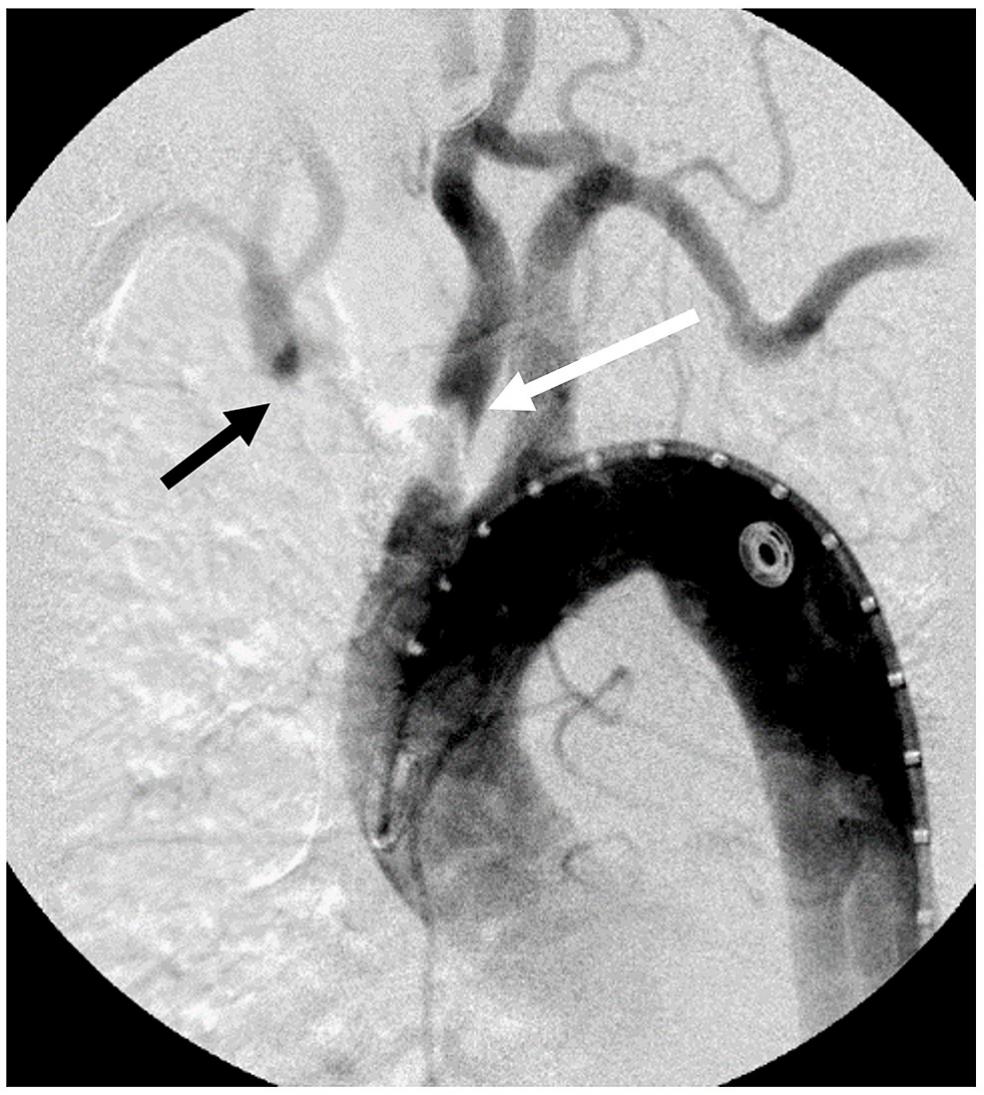
Arch aortogram demonstrating complete occlusion of the innominate artery (black arrow) with delayed filling and severe stenosis of the left common carotid artery (white arrow).

Arch aortogram confirmed a heavily calcified occlusive lesion of the innominate artery with significantly delayed retrograde filling as well as severe proximal left common carotid artery stenosis (Figure 3).

Computed tomography angiography (CTA) of the head and neck confirmed an intact circle of Willis but significant reduction of contrast opacification of the right common and internal carotid arteries, right middle cerebral artery, and right vertebral artery.

**Figure 4:**
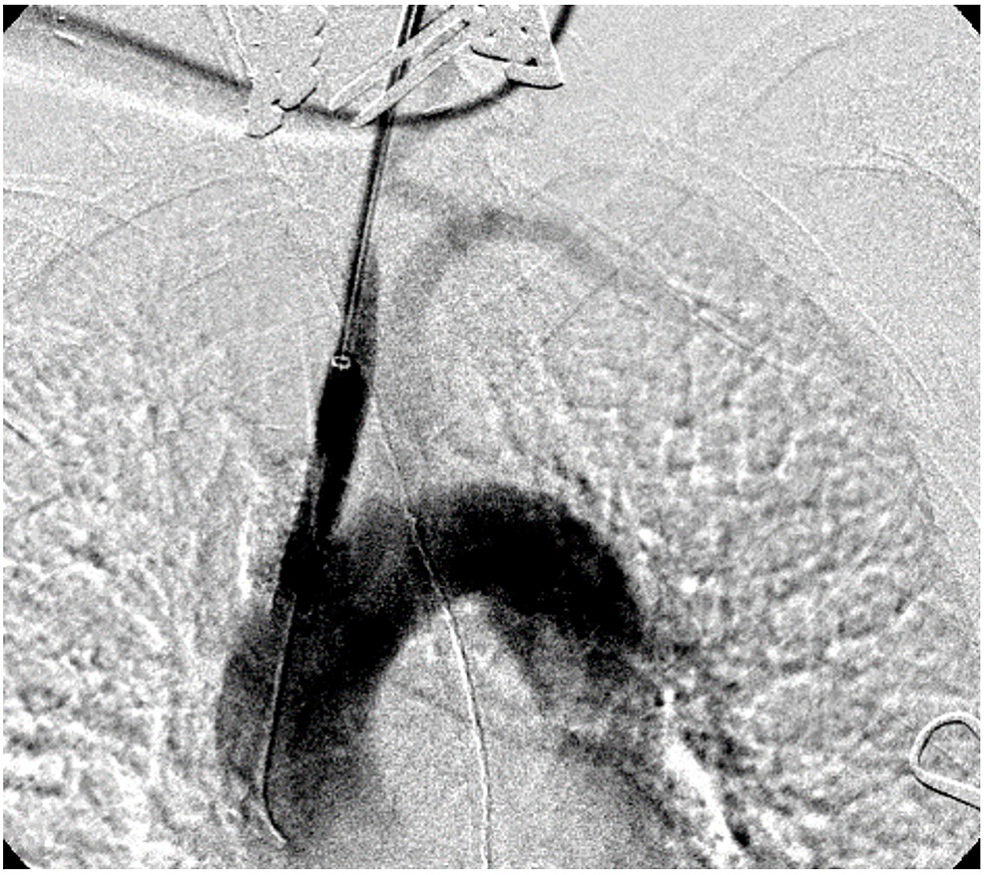
Completion carotid angiogram after stenting demonstrating resolution of the left carotid stenosis. The sheath is within the left common carotid artery in a retrograde fashion after carotid cutdown.

Given the patient’s age and medical comorbidities, bypass originating from the aorta was rendered prohibitively a high-risk procedure. The patient was taken to the operating room for left common carotid to right common carotid artery bypass with stenting of the ostial left common carotid artery lesion. Cerebral oximetry was used to monitor cerebral perfusion throughout the case. The left common carotid artery was exposed and control was obtained. The patient was heparinized and systolic blood pressure was maintained at >160 mmHg. The left carotid artery was accessed in a retrograde fashion. The left common carotid artery was then clamped distal to the sheath. The lesion was then crossed with a glidewire over which a sheath was advanced beyond the lesion. The dilator was removed and a 7 × 39 mm Viabahn balloon-expandable (VBX) stent graft (W.L. Gore & Associates, Inc., Flagstaff, AZ) was advanced so that the entire lesion was crossed with the most proximal part of the stent just inside the aortic arch. The sheath was retracted, and the stent deployed. After placement, the angiogram demonstrated the resolution of the stenosis (Figure 4).

The sheath was removed. Forward and back bleeding allowed for flushing of debris. The access arteriotomy site was extended and used as the proximal anastomosis site for a 6 mm heparin-bonded ring reinforced prosthetic graft. After anastomosis was completed, the distal common carotid clamp was released and the graft was clamped, allowing the return of antegrade flow through the left common carotid artery. The right common carotid artery was then exposed. A retropharyngeal tunnel could not be safely created given the patient’s prior extensive oropharyngeal resection and reconstruction, so a pretracheal tunnel was created. The graft was passed through the tunnel, and an anastomosis was created to the right common carotid artery. The patient tolerated the procedure well with no neurologic deficits and had an uncomplicated postoperative course. She was discharged home on dual antiplatelet therapy on postoperative day two.

Follow-up

**Figure 5:**
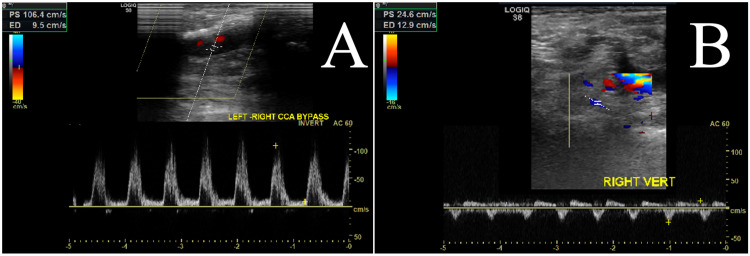
Follow-up duplex imaging shows a patent left carotid to right carotid artery bypass graft (A) and oscillation of flow in the right vertebral artery (B).

Postoperative duplex at one-month and six-month follow-ups showed a patent carotid artery bypass graft (Figure 5A) and oscillation of flow in the right vertebral artery (Figure 5B).

At one-year follow-up, the patient continued to have complete resolution of symptoms, and duplex surveillance demonstrated a patent carotid-carotid bypass graft.

## Discussion

Anatomic considerations are key to the treatment of innominate artery disease. The prominent central location of the innominate artery along with the slow progression of occlusive lesions allows the development of robust collaterals. The right vertebral artery may have blood supply from the contralateral vertebral artery, ipsilateral external carotid artery branches, and the circle of Willis via posterior communicating arteries. The right internal carotid artery may receive blood from the external carotid artery collaterals as well. The abundant collateral network of the vertebral and internal carotid arteries likely explains the discrepancy between angiographically significant stenosis and normal duplex waveforms. Although right common carotid artery flow remained cephalad in this case, the flow was chronically diminished as evidenced by the presence of clinical symptoms and delayed opacification of the right carotid artery and its branches on angiography.

Symptomatic complete occlusions of the innominate artery may require surgical bypass either through traditional sternotomy or mini-sternotomy [7]; however, this requires at least partial clamping of the ascending aorta. Mortality of transthoracic open surgery for innominate artery disease varies from 3% to 16% [8,9] with perioperative stroke rates of up to 6% [10]. Given this patient’s age and medical comorbidities, she was not a candidate for an open thoracic bypass procedure.

Extrathoracic bypass procedures portend lower mortality but have a high complication rate of 15% to 25% [8,9]. Thus, these operations are typically reserved for failure of endovascular techniques. Endovascular procedures are now considered the first-line therapy for supra-aortic trunk lesions [11-13]. Stenosis of the innominate artery can often be treated with endovascular interventions; however, three-year restenosis rates are as high as 32% [14,15]. Innominate artery stenting can be complicated by stent fracture, restenosis, and migration [16-18]. Stroke rates following innominate artery stenting range between 2% and 11% [13,19].

Retrograde stenting of the innominate artery has been described with cerebral protection achieved through distal clamping after direct cutdown of the right common carotid artery [20]; however, the occlusive lesion in the case presented was heavily calcified and relatively long. A left carotid artery to right carotid artery bypass circumvented the risks associated with crossing and stenting this calcified long segment of innominate artery occlusion. Stenting of the left common carotid artery lesion was required to optimize inflow into the bypass. This was performed in a retrograde fashion to provide embolic protection. Furthermore, the technique of clamping the distal common carotid artery only when crossing the lesion and moving the clamp to the graft after construction of the anastomosis significantly reduced total clamp time.

## Conclusions

Innominate artery occlusion with severe intrathoracic left common carotid artery stenosis presents a unique challenge. A hybrid treatment with retrograde left common carotid artery stenting and left to right common carotid artery bypass is described. Informed consent has been obtained from the patient for publication of the case report and accompanying images.
